# Cluster headache: an overview of established and emerging treatments

**DOI:** 10.1007/s00415-019-09548-x

**Published:** 2019-10-10

**Authors:** A. Al-Ansari, N. P. Robertson

**Affiliations:** 1 Department of Neurology, University Hospital of Wales, Heath Park, Cardiff, C14 4XN UK; 2 Department of Neurology, Institute of Psychological Medicine and Clinical Neurosciences, Cardiff University, Cardiff, C14 4XW UK

## Introduction

Cluster headache is a rare but disabling neurological disorder, characterised, according to the International Classification of Headache Disorders-3 (ICHD-3), by unilateral severe headache with associated autonomic features and agitation. Despite proven abortive and preventive treatments, many cases remain refractory, leading to adverse psychosocial and employment outcomes. Understanding the efficacy of current and emerging therapies may help to improve clinical management of such cases.

This month’s journal club reviews three papers relating to treatment of cluster headache. The first explores the effectiveness and adverse effects of a range of acute treatments for cluster headache, based on a large international sample. The second is a pooled analysis of two previous trials evaluating non-invasive vagus nerve stimulation (nVNS) as an acute treatment for cluster headache. The third paper describes a double-blind, randomised, placebo-controlled study assessing the monoclonal antibody galcanezumab as a preventive agent for cluster headache.

## Effectiveness of oxygen and other acute treatments for cluster headache: results from the cluster headache questionnaire, an international survey

Data was collected over a two-year period via the Cluster Headache Questionnaire which was available as an internet-based, self-administered survey in several countries. The majority of respondents were from the United States, United Kingdom and Canada. The questionnaire was advertised on headache society websites and via email to society members. The questionnaire comprised 152 items within 8 sub-sections including consent and personal details. A further section also addressed the ICHD-3 criteria for diagnosis of cluster headache and enabled exclusion of those without this diagnosis. The remaining sections addressed effectiveness of medication, physical and psychological complications of treatments, mood scores and difficulties obtaining medication.

A range of statistical tests analysed and compared the effectiveness of acute treatments in participants with diagnoses of definite or probable cluster headache and in respondents over the age of 65.

A total of 2193 participants met criteria for the study (1604 cluster headache and 589 probable cluster headache). Fifty-four per cent of all respondents reported complete or very effective response to oxygen and triptans, with lower efficacy for other acute treatments: dihydroergotamine (25%), ergotamine (17%), intranasal ketamine (14%), caffeine and energy drinks (17%), opioids (6%), intranasal capsaicin (5%), intranasal lidocaine (2%). Adverse effects in the form of physical or emotional complications were lowest for oxygen, lidocaine and ketamine, but higher for ergot derivatives, opioids and triptans. Responses in the over 65 age group for treatment efficacy and adverse effect profile were similar to the combined cohort. Patients with episodic cluster headache responded better to oxygen than those with chronic cluster headache, although there was no difference in response between episodic and chronic cluster headache to triptans.

Further analysis revealed difficulty obtaining oxygen as compared to other treatments, with reasons cited including practicalities, the respondent’s smoking status and issues relating to insurance cover.

**Comment:** This large international sample demonstrates the high efficacy and minimal complication rate of oxygen as an acute treatment for cluster headache, with similar efficacy for triptans but with a higher adverse effect profile. The poor efficacy and high side effect profile of opioid treatments is also noted. Strengths of this study include the large sample number and the strict inclusion of ICHD-3 criteria. Limitations include the lack of validation of the questionnaire prior to the study. Self-administration introduces subjectivity and recall bias, with no verification from the respondents’ medical notes or practitioner. The questionnaire also did not allow respondents to elaborate on the nature of adverse effects. Finally, the questionnaire did not include indomethacin or steroids as acute treatments despite their wide use in autonomic cephalalgias.

*Pearson et al. Headache 2019 Feb;59(2):235–249*

## Differential efficacy of non-invasive vagus nerve stimulation for the acute treatment of episodic and chronic cluster headache: a meta-analysis

This study is a meta-analysis of two randomised, double-blind, sham controlled trials (ACT1, ACT2) which evaluated nVNS as an acute treatment in episodic and chronic cluster headache. The similarity between the study designs and populations of the two trials allowed pooling of data and enabled greater statistical power in the analysis of results.

225 participants with episodic (*n* = 112) and chronic (* n* = 113) cluster headaches were included from both data sets. The main outcome measures were the primary endpoints of each study. Logistic regression models were used to analyse the proportion of participants whose first treated attack had improved from pain intensity 2–4 to 0–1 at 15 min after treatment initiation, and the proportion of participants in whom ≥ 50% of all treated attacks had improved from pain intensity 2–4 to 0–1 at 15 min after treatment initiation.

The study demonstrated that, among participants with episodic cluster headache, more participants treated with nVNS that with sham achieved a statistically significant improvement in their pain intensity in the first treated attack (ACT1 group* p* < 0.01, ACT2 group *p *  = 0.07, pooled *p* < 0.01) and a higher proportion of pain improvement in all treated attacks (ACT1* p* < 0.05, ACT2 *p *< 0.05, pooled *p * < 0.01). Thirty-eight of 124 participants in the nVNS group experienced adverse effects, the most common of which was perioral muscle contraction. Efficacy was not demonstrated for nVNS for chronic cluster headache.

**Comment:** This paper shows that nVNS is an effective acute treatment for episodic cluster headache (*p* < 0.01) with no serious short-term safety concerns. Strengths of this study include the pooling of data from two similar trials to reach significant conclusions. However, there were differences between the two studies, such as variation in stimulation sites, and the allowance of extra pulses in ACT2, which may have accounted for the slight differences in efficacy between the two trials. Possible explanations for the poor response of chronic cluster headache patients to nVNS include higher levels of inter-paroxysmal pain in chronic headache, and differences in brain pharmacology between chronic and episodic cluster headache.

*De Coo et al. Cephalalgia 2019. Vol 39(8) 967–977*

## Trial of galcanezumab in prevention of episodic cluster headache

Cluster headache disease activity is associated with alterations in calcitonin gene–related peptide (CGRP) levels, hence the rationale for evaluation of galcanezumab (a humanized monoclonal antibody to CGRP) for the prevention of cluster headache. One hundred and six participants were enrolled over a 3-year period at 35 sites in Europe and North America. Despite the wide geographical, the planned sample size of 162 was not reached because too few patients met eligibility criteria during the recruitment period. The trial comprised a screening period, a prospective baseline period, and an 8-week double-blind placebo-controlled period. Patients transitioned from the screening period to the prospective baseline period once they had entered an active cluster headache phase. The prospective baseline period involved monitoring of headache attacks over 10–15 days and was used to verify a diagnosis of episodic cluster headache. Patients who met all eligibility criteria were randomly assigned to galcanezumab (300 mg) or placebo once monthly in a double-blind manner. The number and severity of attacks was then monitored over an eight-week period via an electronic patient diary. Patients were further observed over a four-month wash-out period to monitor for side effects.

The mean reduction in the weekly frequency of cluster headache attacks was 8.7 attacks in the galcanezumab group, as compared to 5.2 in the placebo group (*p* = 0.04). The percentage of patients who had a reduction of at least 50% in headache frequency at week 3 was 71% in the galcanezumab group and 53% in the placebo group. There was no substantial difference in the incidence of adverse effects between the groups.

**Comment:** This study demonstrates that subcutaneous galcanezumab reduced the frequency of attacks of episodic cluster headache as compared with placebo. After week 4 of the double-blind assessment period, the frequency of headache attacks between the two groups converged which could reflect the natural remission phase of the cluster headache episode. Limitations of this study include small sample size, and the inclusion of patients with episodic but not chronic cluster headache. Finally, it would be interesting to compare the doses used in this paper for the treatment of cluster headache with those used in the prevention of migraine.

*Goadsby et al. N Eng J Med 2019; 381:132–41*

## Conclusion

The first paper highlights the efficacy and safety of oxygen as a first line, rapidly acting treatment for cluster headache, with reasonable efficacy and side effect profiles for several other agents. The second and third papers demonstrate encouraging progress with neuromodulatory and monoclonal antibody treatments for episodic cluster headache although it remains unclear why many treatments are ineffective in chronic cluster headache. Further work on the pathophysiological differences between episodic and chronic cluster headache could help tailor therapies for chronic cluster headache.

